# Improving the Enzymatic Cascade of Reactions for the Reduction of CO_2_ to CH_3_OH in Water: From Enzymes Immobilization Strategies to Cofactor Regeneration and Cofactor Suppression

**DOI:** 10.3390/molecules27154913

**Published:** 2022-08-02

**Authors:** Carmela Di Spiridione, Michele Aresta, Angela Dibenedetto

**Affiliations:** 1 Department of Chemistry, University of Bari, Campus Universitario, Via Orabona, 4, 70126 Bari, Italy; carmela.dispiridione@uniba.it; 2 Interuniversity Consortium on Chemical Reactivity and Catalysis-CIRCC, Via C. Ulpiani, 27, 70126 Bari, Italy; michele.aresta@ic2r.com; 3 IC2R s.r.l. Via C. Rosalba 49, 70124 Bari, Italy

**Keywords:** biocatalysis, CO_2_ reduction, cofactor regeneration, enzyme immobilization, methanol from CO_2_ and water

## Abstract

The need to decrease the concentration of CO_2_ in the atmosphere has led to the search for strategies to reuse such molecule as a building block for chemicals and materials or a source of carbon for fuels. The enzymatic cascade of reactions that produce the reduction of CO_2_ to methanol seems to be a very attractive way of reusing CO_2_; however, it is still far away from a potential industrial application. In this review, a summary was made of all the advances that have been made in research on such a process, particularly on two salient points: enzyme immobilization and cofactor regeneration. A brief overview of the process is initially given, with a focus on the enzymes and the cofactor, followed by a discussion of all the advances that have been made in research, on the two salient points reported above. In particular, the enzymatic regeneration of NADH is compared to the chemical, electrochemical, and photochemical conversion of NAD^+^ into NADH. The enzymatic regeneration, while being the most used, has several drawbacks in the cost and life of enzymes that suggest attempting alternative solutions. The reduction in the amount of NADH used (by converting CO_2_ electrochemically into formate) or even the substitution of NADH with less expensive mimetic molecules is discussed in the text. Such an approach is part of the attempt made to take stock of the situation and identify the points on which work still needs to be conducted to reach an exploitation level of the entire process.

## 1. Introduction

The continued growth of the world population and standard of life causes an increase in energy consumption and, in a business-as-usual trend, the CO_2_ atmospheric concentration. In fact, fossil carbon (coal, oil, LNG) represents 81+ % of the primary energy sources actually used. The putative effect is climate change and potential extreme events that may cause danger to human life. It is urgent to reduce the number of greenhouse gases (GHGs) in the atmosphere, as stated in the IPCC 2022 Report [1] and at the Glasgow COP 2021 [2]. The use of C-free primary energy sources and the capture and conversion of CO_2_ into value-added products contribute to reaching the goal [3,4,5]. The process of CO_2_ capture and utilization is part of the larger concept of Circular Economy, whereby CO_2_ becomes a resource from which to obtain added-value commercial products [6,7]. Chemical, electrochemical, photochemical, and enzymatic routes are being investigated to achieve this goal.

**Figure 1:**
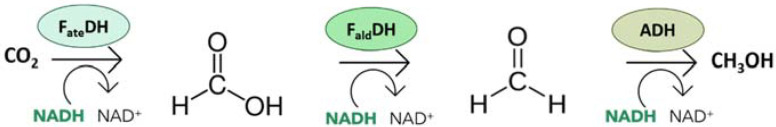
Cascade of reactions for the reduction of CO_2_ to CH_3_OH.

An attractive case of CO_2_ utilization is the biotechnological reduction of CO_2_ to methanol [8,9,10,11,12] (Figure 1), which has multiple uses as a raw chemical and fuel. This reaction has aroused much interest, and in recent years, several research groups have tried to take stock of much research efforts: reviews describing particular aspects of the reaction condition can be found in the literature [13,14,15]. 

The main interest in this cascade of reactions lies in the mild reaction conditions. As a matter of fact, the conversion of CO_2_ to methanol occurs in water at room temperature and is assisted by three dehydrogenases, specifically: *Formate dehydrogenase* (*F_ate_DH*), *Formaldehyde dehydrogenase* (*F_ald_DH*), and *Alcohol dehydrogenase* (*ADH*). Such enzymes use the cofactor nicotinamide adenine dinucleotide (NADH) as an energy provider. The stoichiometry of the 6e− reduction–reaction is driven by the cofactor: three moles of NADH are oxidized to NAD^+^ and produce one mole of CH_3_OH (Figure 1). Since the limiting agent is NADH, the latter is used to calculate the reaction yield (Equation (1)) [8].

Y% = [(3xmol CH_3_OH)/mol NADH] × 100) (1)

Even though the cascade of reactions has been known and studied for a long time, strategies are still being sought to overcome several limitations. In fact, among the disadvantages of such a reaction, one can list i. the high costs and rapid deactivation of the enzymes; ii. poor CO_2_ solubility in water; and above all, iii. the high cost of the cofactor, which is also an unstable species and has to be used in large excess to drive the reaction. All these impediments make the reaction yield very low (free enzyme system yield = 10–20%) and not applicable on a large scale. To overcome such limitations, researchers over the years have started to think about ways to:

- Make enzymes more robust, i.e., methods of immobilization that also facilitate enzymes recovery and recycling;
- Increase the availability of CO_2_ in solution either by adding specific enzymes that drive the hydration/dehydration process or by using materials capable of capturing CO_2_;
- In situ and non-in situ methods that allow selective regeneration of the cofactor or even reduce or eliminate the cofactor itself, using artificial cofactors or adopting a direct electron transfer.

The purpose of this review was to provide a comprehensive overview of the advances made to date to overcome the above limitations and to indicate what appear to be the most interesting prospects for future studies.

## 2. The *Dehydrogenases*

The three dehydrogenase enzymes involved in the CO_2_ to methanol conversion are: *Formate dehydrogenase*, *Formaldehyde dehydrogenase* and *Alcohol dehydrogenase*. Dehydrogenases are enzymes that catalyze the “proton coupled to electron transfer” (PCET, H^+^ + e^−^) from a molecule that acts as “electron + proton” donor (reductant) to another one that acts as an acceptor (oxidant). During the redox reaction, NADPH/NADP^+^ or NADH/NAD^+^ is employed as an essential cofactor. The three enzymes above manage the reduction in the oxidation state of carbon dioxide to carbon-based energy-carrier resources [14].

### 2.1. Formate Dehydrogenase

*Formate dehydrogenases* are categorized as metal-independent and metal-dependent based on the presence of metals (molybdenum-Mo or tungsten-W) in the active sites. The former catalyzes the reaction from HCO_2_H to CO_2_ irreversibly; the latter catalyzes the reduction of CO_2_ to HCO_2_H reversibly and, because of this, is also employed in the enzymatic CO_2_ to HCO_2_H conversion [13]. The redox potential for the enzymatic reduction of CO_2_ to HCO_2_^−^ is E°ʹ = −420 mV, but both types of *F_ate_DHs* mainly catalyze HCO_2_^−^ oxidation, hence their dehydrogenase designation. The difference in catalytic reaction reversibility is due to differences in the catalytic reaction mechanism and the enzyme structure governing the energy reorganization during catalysis [16]. Among these *F_ate_DHs*, the one extracted from *Candida boidinii* (EC 1.2.1.2, *CbF_ate_DH*) is commercially available and can be easily handled as a catalyst for CO_2_ reduction. *CbF_ate_DH* is a homodimer [79 kDa (6 nm × 6 nm × 10 nm)] with two independent active sites catalyzing the NAD^+^-dependent oxidation of formate to CO_2_ via an irreversible hydride transfer from formate to NAD^+^ [17].

The conversion of CO_2_ to formate is a process thermodynamically unfavorable and which somehow needs to be “helped” to happen. Table 1 shows the *Km* values with reference to the substrates of the reaction in the two possible directions. The *Km* is the index of the affinity between enzyme and substrate: the lower its value, the higher the affinity of the substrate for the enzyme. As reported in Table 1, such value is lower for CO_2_ than for HCO_2_^−^, confirming that the formate oxidation reaction is favored. Nevertheless, the formate reduction reaction can be “forced” to take place by working with an excess of substrate and cofactor, i.e., with high amounts of NADH and CO_2_.

**Figure 2:**
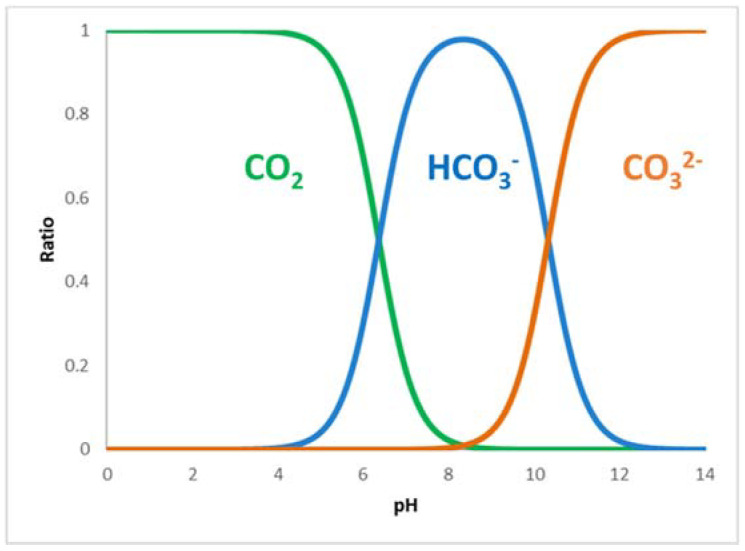
Distribution of species derived from CO_2_ according to pH in water solution [19].

While for NADH, the excess is reached by adding the cofactor in the solution, for CO_2_, it is not so simple due to its low solubility and its interaction with water. Moreover, one must consider that depending on the pH of the solution, CO_2_ in water is found in three forms: carbon dioxide, hydrogencarbonate, and carbonate, as shown in Figure 2. 

There are conflicting opinions in the literature on the role of the CO_2_ hydration reaction. Sato et al. [20] affirmed that as carbonate and hydrogencarbonate concentrations increase in solution, there is a suppression of the formate formation reaction due to the fact that such species are competitive inhibitors of carbon dioxide for the formate production with *CbF_ate_DH*. Other authors, see Wang et al. [21], consider hydrogencarbonate to be the active species, and in some cases (Cazelles et al. [18]), KHCO_3_ is dissolved in water rather than bubbling CO_2_ for better reproducibility. In order to increase the presence of CO_2_ in the solution, this is left to bubble for at least half an hour before starting the reaction, and in some cases, a pressurized system is also used. However, the low solubility of CO_2_ remains one of the reasons for the low yields of such a reaction. In order to improve the yield, a fourth enzyme is added to the system, i.e., *Carbonic anhydrase*, which very quickly (*k_Cat_* = 10^6^ s^−^^1^) catalyzes the hydration–dehydration of CO_2_. (Equation (2))

(2) $${CO}_{2}+H_{2}O\;⇋H^{+}+{HCO}_{3}^{-}$$

According to Wang et al. [21], with the catalysis of *CA*, CO_2_ molecules are rapidly hydrated and transformed into hydrogencarbonate, which is converted into formate, while the cofactor NADH is oxidized. Another possibility to increase CO_2_ availability is to use materials such as MOFs able to both immobilize enzymes and absorb CO_2_.

Although *Formate dehydrogenase* from *C. boidinii* is the most widely used enzyme for testing the reduction reaction, *F_ate_DHs* are also derived from other microorganisms and have been tested for their ability to reduce CO_2_. Nielsen et al. [22] compared the catalytic efficiency of *Formate dehydrogenase* of various organisms by highlighting the type of electron donor, the *Km* for CO_2_, and the working conditions. Amongst the various enzymes, the activity of *DdF_ate_DH* (*Desulfovibrio desulfuricans*) is particularly interesting, with a *K_m_* of 0.02 mM and a catalytic efficiency *K_cat_*/*K_m_* of 2968 [23].

Other *F_ate_DHs* such as, for example, *F_ate_DH* from *Thiobacillus* sp. KNK65MA [24], *F_ate_DH* from *Myceliophthora thermophila* [25], *F_ate_DH* from *Clostridium carboxidivorans* [26], on the other hand, have a low *K_m_* (0.95, 0.44, and 0.05 mM, respectively) but present a not very high catalytic efficiency (*K_cat_*/*K_m_* = 0.34, 0.23, 1.6, respectively).

### 2.2. Formaldehyde Dehydrogenase

The second step of the reaction is the reduction of formate to formaldehyde. The enzyme involved in such a reaction is *Formaldehyde dehydrogenase*. Again, as observed in the first step, the enzyme has more affinity for formaldehyde than formate, and this affects the reaction yield (Table 2). The kinetic parameters of *F_ald_DH* for the reduction reaction (HCO_2_H → HCHO) have not been determined yet, mainly due to the difficulty of measuring the reaction rates at different formic acid concentrations while keeping the pH constant [27]. *Formaldehyde dehydrogenase* (*F_ald_DH*, EC 1.2.1.46) used in the reduction of formic acid is extracted from *Pseudomonas putida* and is a homo-tetramer of approximately 168 kDa [4 × 42 kDa (5 nm × 6 nm × 10 nm)] [18]. 

It was shown that the formate reduction step in the enzymatic cascade from CO_2_ to methanol is the bottleneck of the reaction, as this enzyme has low activity and is sensitive to pH, substrate, and product concentration [18,27,28]. Luo et al. [29] reported that if a minimum concentration of 10 mM of formate is not reached, the reaction does not proceed, and higher concentrations at the same time do not improve the speed of the reaction. Therefore, they defined 10 mM as the optimum concentration for the reduction to occur. Such information is obtained by a step-by-step study of the cascade reaction. Given this, it is possible to state that in the cascade reaction, the slow accumulation of formate affects the low yield of the second reduction step from formate to formaldehyde. Starting from formate instead of CO_2_ would, thus, help the process.

### 2.3. Alcohol Dehydrogenase

The third enzyme involved in this reaction is *Alcohol dehydrogenase*. *Alcohol dehydrogenase* is present in many organisms, but the mainly used is the one from *Saccharomyces cerevisiae* (*ADH*, EC 1.1.1.1, a homo tetramer of about 141–151 kDa with a size of 7 nm × 10 nm × 11 nm), which is commercially available. *ADH* normally acts on primary or secondary alcohols, but the enzyme oxidizes methanol much more poorly than ethanol. In the cascade reaction, it is used in the final step of the multienzyme process, i.e., the reversible reduction of formaldehyde to methanol [30]. The forward reaction (formaldehyde → methanol) is much more efficient than the reverse one (methanol → formaldehyde) given the affinity of the substrates for the enzyme (see the km value in Table 3); the reduction in formaldehyde is considered almost irreversible. This step is the only one favored in the direction of the cascade reaction from CO_2_ to methanol. However, *ADH* is not a very stable enzyme for industrial use. Indeed, when the three dehydrogenases catalyzing the cascade reaction from CO_2_ to CH_3_OH were employed as free enzymes, *ADH* was by far the least stable one [31].

## 3. Study of Reaction Conditions

In such a cascade of reactions, the reaction conditions must be balanced to best suit the optimum work rates of the three enzymes simultaneously. Reaction conditions concern pH, temperature, and CO_2_ pressure. Moreover, the amount of cofactor and the ratio between enzymes have been studied over time and optimized.

*F_ate_DH* and *F_ald_DH* have similar working optimums (pH 6–7, 37 °C), and the fact that both enzymes work most efficiently under similar conditions is essential for the cascade reaction to being effective. Those conditions, however, are not favorable for the third enzyme in the system, *ADH*, which works best under somewhat different conditions, pH 8–9 and 25 °C [27]. It was observed that *F_ate_DH* and *F_ald_DH* could not reduce CO_2_ and HCO_2_^−^, respectively, at pH > 8.5. According to the literature, the overall reduction of CO_2_ to methanol should be carried out at pH values between 6 and 7 to allow at least 80% efficiency of each enzyme [18]. Generally, there is a tendency to prefer conditions that favor the activity of the first two enzymes over the third, mainly because the first two enzymes are more unlikely to allow the reaction to take place due to thermodynamics. The reaction is generally conducted at 37 °C with a pH ranging between 6.5 and 7: the pH is kept stable by using buffer solutions of Tris-HCl (tris(hydroxymethyl)aminomethane hydrochloride) or PBS (Phosphate buffered saline), as required. 

Another fundamental factor defining the reaction conditions is the amount of NADH. For reactions to take place, it is necessary to work with an excess of NADH so that the reaction can be directed to the right, as reported in the literature. NADH is a highly unstable species, sensitive to temperature, pH, and buffers, the last without appreciable interactive effects. The UV spectrum of the cofactor (reduced form) was monitored at 340 nm, and the decomposition was followed by monitoring the absorption peak signal at such wavelength. Only the reduced form absorbs at 340 nm, while the oxidized form does not: the decomposition of NADH or its oxidation can be advantageously studied by following the absorbance at this wavelength. NADH decomposition can be observed both at low pH < 4 and high pH > 8. The study of NADH decomposition in various buffers shows that there is greater decomposition in the presence of PBS [32,33]. In order to avoid the natural oxidation of NADH due to the presence of oxygen in the air, the reactions are conducted in a controlled atmosphere of nitrogen or carbon dioxide. A recent study also highlighted the inhibitory effect of NADH concentration on enzymes and, in particular, *F_ate_DH*. In fact, the study shows that the activity of the enzyme, whether it is free or immobilized, drops from 100% if the NADH concentration is 0.45–0.55 mM, respectively, for the free and immobilized enzyme, to 60% for the free enzyme already at a concentration of 0.52 mM and 80% for the enzyme immobilized with NADH 0.61 mM [34,35,36].

As said above, in order to increase the amount of CO_2_ in the solution, its pressure can be increased. From past studies [18,37,38], it was established that the best pressure condition is 0.5 MPa of CO_2_, usually preceded by bubbling the CO_2_ to saturate the solution. 

In the end, the ratio of enzymes is also a parameter to be considered. Studies by Cazelles et al. [18] and Nabavi Zadeh et al. [27] showed that the best ratio between the enzymes is 1:15:75. These studies were conducted by studying two enzymes at a time, keeping the concentration of one constant, and changing that of the other. Despite such findings, in many articles, the ratio between enzymes is kept at 1:1:1. This is an important parameter but secondary to those discussed above because it can be changed after fine-tuning the system.

### 3.1. Enzymes Immobilization and Co-Immobilization

From the perspective of recycling enzymes to reduce production costs, it is important to think of a way to make the enzyme more easily recoverable from the reaction solution while assuring a longer life. The immobilization of an enzyme makes it a heterogeneous catalyst that can be efficiently separated. Immobilization not only makes the enzyme “separable” from the reaction mixture and thus reusable but could improve its stability, activity, and resistance to inhibitors. One reason could be found in the fact that the enzyme could be less in contact with: solvent, possible inhibitors, and in general, cannot interact with external interfaces. However, this always depends on the immobilization mode used, which can make the enzyme more or less accessible. For example, immobilization on the external surface of a support does not give the same protection as an enzyme immobilized inside a support, thus covered by the same [39]. Furthermore, the properties of the support, both physical and chemical, may or may not facilitate the activity of the enzyme. In general, the support should be as inert as possible. However, in some cases, the support can become an integral part of the process itself; for example, it could, depending on its characteristics, make the substrate more accessible to the enzyme or, on the contrary, it could displace the product, and this could be interesting since there are enzymes that suffer from product inhibition, including *Formaldehyde dehydrogenase* [40].

When choosing the support, one must think of something that must be optimal for the enhancement of the enzyme’s catalytic capacity. Not all supports may work well with all enzymes. In order to boost the enzyme activity, it is necessary to design and select the best supports for their particle size, diverse morphological shapes, and lower diffusional restrictions to yield high-value chemical products. Additionally, supports must have these enticing peculiarities such as high porosity, large surface area, inertness, physical firmness, resistance to microbial attack, the correct density of functional groups, and regenerability to guarantee the best performance of the biocatalyst [32].

Another aspect to be assessed concerns the conformational changes that the enzyme may undergo following immobilization. Conformational changes in enzymes may be necessary for the fulfillment of their function, and the assumption of certain specific forms can improve the stability of the enzyme itself, so if immobilization allows these conformations to be maintained, the enzyme will be more stable. In other cases, the conformational changes may be such that they lead to partial or total denaturation of the enzyme, resulting in lowered activity or complete inactivation. Very often, the deactivation of enzymes is due to structural changes in the enzymes, especially if they have several subunits that could dissociate; the choice of an appropriate method of immobilization has to take this into account, e.g., it may be necessary to choose a method that allows all subunits to interact with the substrate so that they cannot dissociate [40,41].

In addition, the immobilization of enzymes on the support influences their activity, making it difficult to determine the activity of individual enzymes.

A key point in the case of cascade reactions is the choice between immobilization and co-immobilization. Although co-immobilization may initially appear to be the best choice because it brings advantages with respect to the proximity of enzymes to each other and thus also to the products that are to become substrates for subsequent enzymes, it is a choice that requires special analysis and attention. Perfect co-immobilization of enzymes is very difficult to achieve, they may have different stability and size and prefer different immobilization conditions, but to be immobilized at the same time, it is necessary to narrow down the range of conditions that can be changed to suit all the enzymes involved. The full benefit of using immobilization is achieved if it simultaneously improves the properties of all enzymes and an optimal immobilization protocol identical for all is identified [42,43].

The conversion of CO_2_ to CH_3_OH is a classical cascade of reactions where the product of one enzyme is the substrate of the other enzyme, whose product is the substrate of another enzyme. Table 4 shows the most representative works in the literature regarding the method used to date for immobilization of single enzymes or co-immobilization of the enzymes in the cascade from CO_2_ to CH_3_OH. In such a reaction, the use of co-immobilized enzymes has a kinetic advantage. As the reactions from CO_2_ to HCO_2_^−^ and from HCO_2_^−^ to HCOH tend to turn back, immobilizing the three *dehydrogenases* within a finite area reduces the diffusion path of the intermediate products (HCO_2_H and HCOH) to the next enzyme’s active site, which hypothetically would increase the conversion rate, avoiding the back reaction [27]. On the contrary, such immediate conversion also maintains the substrate levels at a very low concentration, which lowers the individual enzymatic rates [13].

Co-immobilization becomes even more interesting if one also considers co-immobilizing the cofactor, as it permits the reuse of the cofactor molecules by several reaction cycles [40,44].

**Table 4:** Enzymes immobilization methods and bibliographic key outcome.

Immobilization Matrix	Immobilized Enzymes	Note/Key Outcome	Ref.
SiO_2_ sol–gel	*F_ate_DH, F_ald_DH, ADH*	Yield-free enzymes = 10–20% Yield-immobilized enzymes = 40–90%	[45]
SiO_2_ sol–gel	*F_ate_DH, F_ald_DH, ADH*	Yield-free enzymes = 98.1% Yield-immobilized enzymes = 92.1%	[46]
ALG-SiO_2_, hybrid gel	*F_ate_DH, F_ald_DH, ADH*	Yield-free enzymes = 98.8% Yield-immobilized enzymes = 98.1%	[47]
PS NPs	*F_ate_DH, F_ald_DH, ADH*	Yield-free enzymes = 12%. Yield-immobilized enzymes = 127% (80% initial activity retained after 11 cycles). Enzymatic regeneration with *GDH*	[48]
Capsules-in-bead scaffold	*F_ate_DH, F_ald_DH, ADH*	Immobilized enzymes were more active than free enzymes when a free cofactor was presented.	[49]
Titania–protamine particles	*F_ate_DH, F_ald_DH, ADH*	Yield-free enzymes = 5–10%. Yield immobilized enzymes = 35–60% (50% initial activity retained after 10 cycles)	[50]
ALG-SiO_2_, hybrid gel	*F_ate_DH, F_ald_DH, ADH*	Yield-immobilized enzymes = 100% (external reg.) Yield-immobilized enzymes = 80% (with in situ reg.) Chemical regeneration with SDT	[8]
Phospholipids–silica nanocapsules	*F_ate_DH, F_ald_DH, ADH*	Free enzymes = 0.06 mmol MeOH/g_enzyme_. Immobilized enzymes = 0.88 mmol MeOH/g_enzyme_ Free with *PtDH* = 0.16 mmol MeOH/g_enzyme_ Immobilized enzyme with *PtDH* = 4.30 mmol MeOH/g_enzyme_. Enzymatic regeneration with *PtDH*	[18]
Hybrid microcapsules	*F_ate_DH, F_ald_DH, ADH*	Yield free enzymes = 35.5%. Yield immobilized enzymes = 71.6% (52.6% initial activity retained after 9 cycles)	[21]
Flat-sheet polymeric membranes	*F_ate_DH, F_ald_DH, ADH*	Free enzymes: [MeOH] = 0.5 mM Co-immobilized enzymes: [MeOH] = 0.6 mM mMSeq-immobilized enzymes: [MeOH] = 0.7 mM Enzymatic regeneration with *GDH* and glutamate	[29]
CF electrode with alginate matrix	*F_ate_DH, F_ald_DH, ADH*	Electrochemical CO_2_ reduction to methanol around 0.15 ppm. Faradaic efficiencies of around 10%. No NADH but direct electron transfer	[51]
PS nanofibrous membrane	*F_ate_DH*	Free enzymes: [Formate] = 0.6 mM Immobilized enzymes: [Formate] = 0.3 mM (53% initial activity retained after 8 cycles) Electrochemical regeneration	[34]
Magnetic NPs	*F_ate_DH, F_ald_DH, ADH*	Stepwise scheme led to only a 2.3% yield of methanol per NADH; batch system under CO_2_ pressure, the combination of the four immobilized enzymes increased the methanol yield by 64-fold	[52]
ZIF-8 entrapped in PVDF microporous asymmetric membrane	*F_ate_DH, F_ald_DH, ADH*	Immobilized enzymes without membrane (EMS) = 5 µmol. Immobilized enzymes with membrane (ECMS) = 6 µmol. Disord. Immobilized enzymes with membrane (DEMM) = 7 µmol. Ord. Immobilized enzymes + NADH without membrane (OEMM) = 13 µmol. Ord. Immobilized enzymes + NADH with membrane (OECMM) = 14 µmol. Over 50 % of their original productivity was retained after 12 h of use	[53]
Titania NPs	*ADH*	The results revealed that immobilization of enzymes led to higher catalytic. The activity of *ADH* from 30% to more than 80% of its initial activity after 30 days of storage at 4 °C. (84% initial activity retained after 10 cycles)	[54]
MOF, NU-1006	*F_ate_DH*	Immobilized Enzyme + cofactor Rh: [Formic acid] = 144 mM. Photochemical regeneration with Rh complex	[55]
Zeolite particles	*F_ate_DH*	Yield imm. Enzyme = 34–37%	[56]
MOF, ZIF-8	*F_ate_DH*	Compared with the free multienzyme system, formate yield was increased by 4.6-fold. Co-immobilized with CA and enzymatic regeneration with *GDH*	[57]
Graphene + CF electrode with alginate matrix	*F_ate_DH, F_ald_DH, ADH*	Electrochemical CO_2_ reduction to methanol around 20 ppm. Faradaic efficiencies of around 12%. No NADH but direct electron transfer	[58]
MOF, ZIF-8	*F_ate_DH, F_ald_DH, ADH*	Free enzymes: [MeOH] = 0.061 mM. Immobilized enzymes: [MeOH] = 0.320 mM. Immobilized enzymes + NADH regeneration: [MeOH] = 0.742 mM. Electrochemical regeneration with Rh complex-grafted electrode	[59]
MCF	*F_ate_DH, F_ald_DH, ADH*	Catalytic activity-free enzyme systems = 0.3 mmol MeOH/g_enzyme_ min. Catalytic activity immobilized enzymes systems = 1.35 mmol MeOH/g_enzyme_ min	[60]
Gold and graphite electrodes	*F_ate_DH*	Electrochemical CO_2_ reduction imm. enzyme: [Formate] = 3.7 µM. Faradaic efficiencies of around 100% No NADH but direct electron transfer	[61]

It is not easy to compare the efficiency of immobilization methods because the reactions take place under different conditions in terms of time, reactor type and substrates, and amounts of immobilized enzymes and cofactors used, which make it difficult to identify one method that may actually be better than others. Additionally, it is usually difficult to assess the absolute amount of enzymes available for the substrates when several enzymes have been immobilized simultaneously, which in turn makes it difficult to determine the actual biocatalytic productivity [13]. However, all methods may be considered valid because when comparing the reaction with free enzymes, all methods give better or equal yields with the possibility of recycling. The yields in Table 4 depend on the amount of initial NADH used; they are calculated according to Equation (1) and, in some cases, can reach values higher than 100 percent due to the combination of regeneration of NADH. In the literature, the reaction conditions and the quantities of reagents used are not always well described, so where it was not possible to determine the yield as a percentage, the most interesting data that give an idea of the efficiency of the method used were included. Among the methods of immobilizing the three dehydrogenases, the most widely used in the past is the encapsulation in silica sol–gel [45] or alginate–silica hybrid matrices [8,47]: the observed improvement in methanol yield is certainly due to the confinement of enzymes in a more limited space.

In recent years, research has moved towards more innovative materials such as MOFs (metal–organic frameworks). They were even identified as materials capable of absorbing CO_2_, and this led to an interest in the possibility of using them for the immobilization of enzymes. Among their main characteristics are their large surface area, porosity, and thermal stability. A particularly interesting case is the study by Zhang et al. [59], where the enzymes are immobilized on MOF ZIF-8, and the encapsulation of the enzymes occurs simultaneously with the synthesis of MOF itself. This is possible due to the mild conditions of MOF synthesis and leads to a firm binding of the enzymes to the support. Moreover, the absorption of CO_2_ in ZIF-8 increases its solubility compared to that in water by a factor of 10.

Zhu et al. [53] identified an even better advantage by using co-immobilization in ZIF-8 of enzymes and cofactor by complementing the recycling of the cofactor with its reuse.

Other special cases are those in which enzymes are immobilized on electrodes, for example, the experiments carried out by Schlager [51], Seelajaroen [58], and Alvarez-Malmagro [61]. The interest in the latter type of support stems from the idea of eliminating the need for a cofactor which, as discussed above, is a limiting factor in the reaction. This brings us to the next section: the need to regenerate the cofactor.

### 3.2. Cofactor Regeneration

One of the disadvantages of the cascade reaction is the high cost of the cofactor NADH; for this reason, intensive research was conducted on NADH regeneration, including the enzymatic, chemical, photochemical, and electrochemical approaches.

Representative examples for each regeneration method are summarized in Table 5. Even in this case, it is difficult to make a real comparison between the methods and define the best case because the reaction conditions are different. In some cases, regeneration is tested on its own; in others, it is combined with CO_2_ reduction, either complete to methanol or partial to formic acid or formaldehyde.

#### 3.2.1. Enzymatic Regeneration of the Cofactor

**Figure 3:**
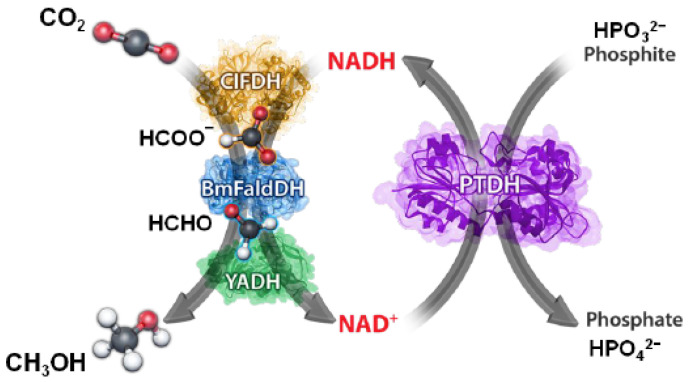
Schematic illustration of CO_2_ reduction coupled to enzymatic cofactor regeneration. Reprinted with permission from ref. [64]. Copyright © 2022 American Chemical Society.

Enzymatic regeneration appears to be the only one applied on an industrial scale. This method has several advantages starting with compatibility with the target; it is conducted at room temperature and in an aqueous environment and neutral pH, and it also allows high specificity and selectivity towards NADH and low energy consumption. Among the various enzymes, the most widely used enzymes for cofactor regeneration in industrial processes are as follows: *Glutamate dehydrogenase* (*GDH*), which shows the highest activity and stability [62] and is commercially available, inexpensive, and easy to manipulate [63]; and *Formate dehydrogenase* (*F_ate_DH*), which does have a unique feature in generating carbon dioxide (CO_2_) as a gaseous by-product [62]. In addition to *GDH* and *F_ate_DH*, other enzymes were used in the literature. For example (see Table 5), Cazelles et al. [18] and Singh et al. [64] tested the enzyme *Phosphite dehydrogenase* (*PTDH*) (Figure 3), and others used the enzyme *Glucose dehydrogenase* (*GCDH*) or *Xylose dehydrogenase* (*XDH*). In many cases, the NADH yield reaches about 100 %, increasing methanol production by up to +1000% [65]. However, the high cost of enzymes and coenzymes, their instability, and the complexity of product purification are driving the search for other regeneration methods.

#### 3.2.2. Chemical Regeneration of the Cofactor

Chemical regeneration is among the least used methods, so much so that there is no longer any active research on this type of regeneration. The chemical method uses reducing reagents; among them, the one that has been most widely used is sodium dithionite (Na_2_S_2_O_4_), which can manage hydrogen and electron transfer to NAD^+^ in a four-step mechanism [62]. The main problem with such regeneration is that a large amount of water-soluble reducing agent needs to be added, and this leads to the deactivation of the enzymes [8,56].

#### 3.2.3. Photochemical Regeneration

In the photochemical approach, the idea is to copy natural photosynthesis [66]. The application of this type of regeneration requires a photosensitizer, an electron mediator, and an electron donor. The photosensitizer is used for recovering the electron mediators and can be inorganic or organic. Organic photosensitizers exhibit 3–100 times better catalytic activity than inorganic ones, whose synthetic processes are unfortunately often complicated and labor-intensive [62,63].

A Rh--hydride complex is usually used as an electron mediator (as reported in Table 5), while water or triethanolamine (TEOA) are predominantly used as electron donors, even if there are cases where H_2_ or ethylenediaminetetraacetic acid (EDTA) are used. What actually is most changed in the various methods proposed in the literature is the photosensitizer, which, as reported above, can be inorganic or organic and is used for the rehabilitation of electron mediators. Among inorganic photosensitizers, the most used is TiO_2_ which is low cost, stable, and shows good activity. However, its absorption of solar energy is limited by the energy band gap (3.2 eV), and research is aimed to modify titanium oxide to improve the absorption of solar light.

**Figure 4:**
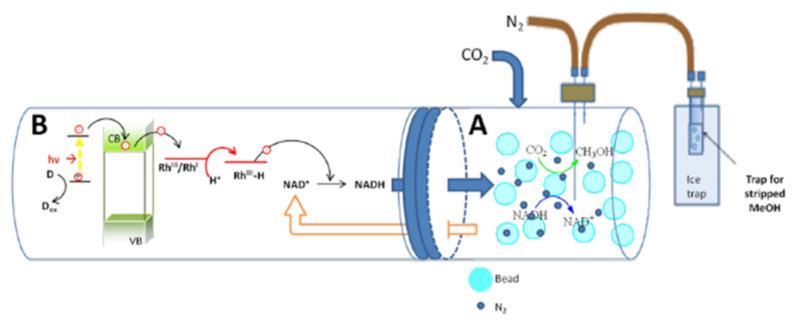
Two-compartment systems for photocatalytic cofactor regeneration in the enzymatic reduction of CO_2_ to CH_3_OH [67].

The result reported by Aresta et al. [67] is interesting, where the addition of a chromium complex and the use of a solution of water and glycerol as electron donors lead to a conversion of NAD^+^ to NADH of up to almost 100% over several cycles (Figure 4).

As far as organic photosensitizers are concerned, among the molecules tested, one finds porphyrins and their derivatives because they structurally resemble chlorophyll and can therefore better mimic photosynthesis. In fact, the porphyrin ring acting as the harvesting site can effectively absorb photons and transfer electrons to the electron mediator. Zhang et al. [68] used an ionic porphyrin for cofactor regeneration, achieving an increase in NADH yield of about 18% with the porphyrin derivative ZnTPyPBr.

This regeneration method has the advantages of being economical and using clean and renewable resources. However, there are some disadvantages because photosensitizers are unstable and difficult to recycle, there is no photocurrent stability, and it is difficult to separate by-products. In addition, long exposure to light can significantly affect enzyme activity unless a two-compartment reactor is used so to use enzymes in the dark and regenerate NADH in the light [67]. The bottleneck here is the different rates of the enzymatic reaction and photochemical regeneration of the cofactor.

#### 3.2.4. Electrochemical Regeneration of the Cofactor

Among the various methods, electrochemical regeneration is the one that could have the most promising large-scale application. This regeneration can be classified as direct, indirect, and indirect enzyme-coupled based on how the electron transfer occurs. In direct regeneration, NAD^+^ reduction occurs in two steps. In the first, there is the formation of the NAD* radical, which is an unstable species and can lead to the formation of dimers (NAD)_2_ that can, in turn, be reduced to form 1,6-NADH, an enzymatically inactive species. If this does not happen, the second step results in protonation of the radical to form 1,4-NADH, which is the active species. Thus, NAD*-radical has two alternative pathways resulting in the production of active 1,4-NADH or inactive 1,6-NADH.

The indirect regeneration, on the other hand, involves the presence of a mediator that causes the reaction to take place in a single step carrying two electrons and one proton, showing higher selectivity than the direct method. The choice of both the electrode potential and material and of the mediator is important for such regeneration because this affects the purity of the product. Barin et al. [34] used a copper foam electrode and a constant potential of −1.1 V, achieving a reduction of NAD^+^ of about 96%; however, the enzyme activity test defining the percentage of active isomer formed established that only about 77% was 1,4-NADH. Kim et al. [69] compared the direct and indirect regeneration mediated by the [Cp*Rh(bpy)Cl]Cl complex, showing how the active form of NADH goes from 67% to 100% using the mediator.

**Figure 5:**
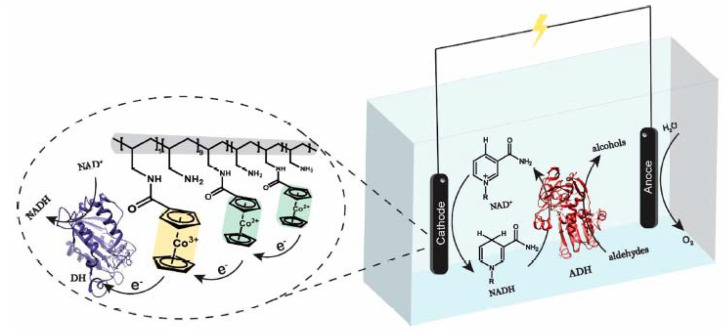
Schematic illustration of electro-enzymatic cofactor regeneration coupled with the reduction of aldehydes to alcohols. Reprinted with permission from Ref. [70]. Copyright © 2022 American Chemical Society.

A particularly interesting case is that studied by Yuan et al. [70], where the third system is used, i.e., the indirect method coupled with enzymes. In this paper, an alternative to the Rh-based mediator that is difficult to remove from the solution and has low turnover frequencies (TOF) is proposed, namely, the use of redox polymers. The authors propose a cobaltocene poly(allylamine) (Cc-PAA) redox polymer where they immobilize a diaphorase for practical NADH regeneration (Figure 5). The result of this regeneration method is extremely interesting as the yield of the active isomer 1,4-NADH is between 97% and 100% [70].

Electrochemical regeneration, compared with the other regeneration methods, has low costs and high efficiency as well as being an environmentally friendly method. However, as with all methods, there are pros and cons. The disadvantages include low selectivity and the high cathode potentials required [71].

**Table 5:** Regeneration methods studied in the literature and their results *.

Regeneration Method	Type of Regenerator	Yield/Key Outcome	Ref.
Enzymatic regeneration	*GDH*	Y_MeOH_ reached 127%	[48]
Enzymatic regeneration	*GDH*	Y_MeOH_ reached up to 95.3%	[72]
Enzymatic regeneration	*PTDH* or *GlyDH*	*PTDH* is 4 times more active than *GlyDH*, [CH_3_OH] increases from 0.1 mM without *PTDH* to 0.9 mM with *PTDH*	[18]
Enzymatic regeneration	*PTDH*	The multienzymatic cascade reaction, along with *PTDH*, yielded 3.28 mM methanol	[64]
Enzymatic regeneration	*GCDH*	Yield of methanol reached 100% after coupling *GCDH* regeneration	[68]
Enzymatic regeneration	*GCDH-XDH*	*XDH* for NADH regeneration was found to be more efficient than *GCDH* producing at least 8 mM CH_3_OH yield	[65]
Enzymatic regeneration	*GDH*	Yield of methanol was increased 64-folds compared to the reaction without a regeneration system	[52]
Enzymatic regeneration	*GDH*	Formate yield was increased 4.6-fold compared to the reaction with free enzymes	[57]
Photochemical regeneration	Carbon-containing TiO_2_/H_2_/[Cp*Rh(bpy)(H_2_O)]^2+^	NADH conversion reaches 94.29% in the presence of H_2_ as an electron’s donor	[73]
Photochemical regeneration	P-doped TiO_2_ nanoparticles/H_2_O/[Cp*Rh(bpy)(H2O)]^2+^	If P to Ti molar ratio is 6%, TiO_2_ nanoparticle can photo catalytically reproduce 34.6% NADH under visible light	[74]
Photochemical regeneration	Cobaloxime/TEOA /eosin	NADH conversion reaches a yield of 36%	[75]
Photochemical regeneration	CCG-IP/TEOA/[Cp*Rh(bpy)(H_2_O)]^2+^	NADH conversion reaches a yield of 38.99% (first cycle) and 36.81% (third cycle)	[76]
Photochemical regeneration	CrF_5_(H_2_O)]_2_−@TiO_2__/_Water-Glycerol/[Cp*Rh(bpy)H_2_O]Cl_2_	NADH conversion reaches the maximum yield (very close to 100%)	[67]
Photochemical regeneration	TiO_2__/_EDTA/[Cp*Rh(bpy)(H_2_O)]^2+^	In the presence of 1.5 mg/mL TiO_2_, the NADH yield reached approximately 90% after 30 min of irradiation	[62]
Photochemical regeneration	ATCN-DSCN/TEOA/[Cp*Rh(bpy)H_2_O]^2+^	NADH yield of ~74%	[77]
Photochemical regeneration	Ionic porphyrin (ZnTPyPBr)/TEOA/[Cp*Rh(bpy)(H_2_O)]^2+^	Yield of NADH increase by 17.9% after 1 h, a seven-fold increase in methanol concentration	[68]
Photochemical regeneration	TiO_2__/_H_2_O/[Cp*Rh(bpy)(H_2_O)]^2+^	Yield of NADH conversion 45.54% (after 2 h)	[78]
Electrochemical regeneration	carbon nanofibers cathode	Yield ~ 99% pure 1,4-NADH	[79]
Electrochemical regeneration	Cu nanorods on glassy carbon	1,4-NADH conversion yield reaches 67%/with electron mediator [Cp*Rh(bpy)Cl]Cl complex reaches almost 100%	[69]
Electrochemical regeneration	Ni NP-MWCNT cathode	Yield ~ 98% pure 1,4-NADH	[80]
Electrochemical regeneration	Cu foam electrode	NADH conversion yield reaches 93–99% 1,4-NADH (active isomer): 75–79%	[34]
Electrochemical regeneration	*DH*/Cc-PAA biocathode	Bioactive 1,4-NADH yield: 97–100% Faradaic efficiencies: 78–99%	[70]
Electrochemical regeneration	Rh modified electrode	NADH conversion yield reaches more than 90% in 20 min	[81]
Electrochemical regeneration	CuNPS on carbon felt electrode	NADH regeneration yield achieves a maximum of 92.1%	[82]
Electrochemical regeneration	Rh complex-grafted electrode	Yield NADH ~ 80% 1,4-NADH reaches almost 100%	[59]

* The key outcome section shows the yield in terms of methanol produced via enzymatic regeneration; for photochemical and electrochemical regeneration, the yield of converted NADH is specified.

### 3.3. Cofactor Substitution

As well as being a highly unstable species, NADH is very expensive, and even if the regeneration can help, this does not resolve the economic problem. In fact, a recycling rate of 99%, if apparently interesting, means that in 100 cycles, the cofactor is lost, and this is not economically acceptable. Since the early years of the study of the cascade of reactions, efforts have been made to synthesize alternative molecules with a structure similar to that of NADH to be able to replace it in the reaction with something simpler to obtain and therefore cheaper. 

**Figure 6:**
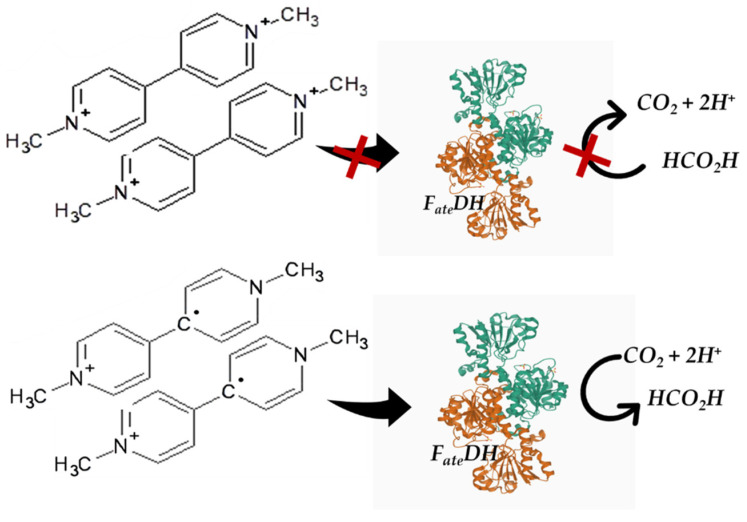
CO_2_ to formic acid conversion using an MV^2+^ cofactor [85].

Nicotinamide cofactor analogs were shown to be essential as NADH models to elucidate structural and mechanistic aspects of enzymatic reactions and useful as a hydride donor or acceptor in redox enzymatic and chemical reactions [83]. An important consideration to make is that using natural cofactors, the reactions of carbon dioxide to formic acid, formic acid to formaldehyde, and formaldehyde to methanol are reversible and ineffective [63]. Furthermore, the catalytic production of CO_2_ from formic acid by *F_ate_DH*, for example, was suppressed using methyl viologen as an artificial coenzyme [84]. Additionally, compared to the natural coenzyme NADH with the analog dithionite reduced methyl viologen, the latter turns out to be a more effective coenzyme in the conversion of CO_2_ to formic acid with *F_ate_DH* (Figure 6) [85].

Amao’s research group devoted much effort to the study and use of such analogs in the CO_2_ reduction reaction [85,86,87]. Among the analogues used are the following molecules: 1,1’-dimethyl-4,4’-bipyridinium salt (methylviologen, MV^2+^); 1,1’-diaminoethyl-4-4’-bipyridinium salt (DA^2+^); 1-methyl-1’-aminoethyl-4,4’-bipyridinium salt (MA^2+^); 1-methyl-1’-carboxymethyl-4,4’-bipyridinium salt (MC^2+^); 1,1’-diacarboxymethyl-4,4’-bipyridinium salt (DC^2+^); 1,1’-dimethyl-2,2’bipyridinium chloride (DM); 1,1’-tetramethykene-2,2’-bipyridinium bromide (QB^2+^); 1,1’-trimethylene-2,2’bipyridinium bromide (TB^2+^); 1,1’-diaminothyl-4,4’-bipyridinium chloride (DAV); 1,1’-ethylene-2,2’-bipyridinium bromide (DB); 1-carbamoylmethyk-1’-methyl-4,4’bipyridinium iodide (CV); 1,1’-dicarbamoylmethyl-4,4’-bipyridinium diiodide (CV), and 1-nicotinamidethyl-1’-methyl-4,4’-bipyridinium salt (NEMBP) [88,89,90,91,92,93].

In 2018, Amao et al. [86] tested MV^2+^ reduced with sodium dithionite for each step of the reaction, determining the affinity of the cofactor for the enzyme by identifying *Km*: their results show that while the affinity for the enzyme *F_ald_DH* cannot be determined, the affinity for the enzymes *F_ate_DH* and *ADH* is the same. Such results identify that the limitation to the use of this cofactor for the CO_2_ to CH_3_OH cascade reaction is its low affinity for the *F_ald_DH* enzyme [86]. In order to improve the reduction efficiency of CO_2_ to formic acid, Ikeyama et al. [88] tried to use methylviolagen derivatives with ionic groups as cofactors, identifying among them that DA^2+^ has a *K_cat_*/*K_m_* value 28 times higher than that of MV^2+^ and 560 times higher than that of NADH and identifying it as a possible substitute for the natural cofactor [88]. The same group tried to modify bipyridines (BPs) with carbamoyl groups and identified CV and CMV as better cofactors than MV. They then verified that modifying the bipyridines with the cationic aminoethyl group to form 1,1’-Diaminoethyl-(DAV) and 1-aminoethyl-1’-methyl-(AMV)-4,4’-bipyridinium salt was more effective compared to the BPs with the anionic carboxymethyl group 1-carboxymethyl-1’-methyl- (CMV) or 1,1’-dicarboxymethyl-4,4’- bipyridinium salt (DCV) [91,94].

Such cofactor analogs have been used for the enzymatic CO_2_ reduction combined with chemical, electrochemical or photochemical regeneration methods. This is because, just as natural cofactors, the analogs exist in both the oxidized and reduced form: in the reaction, they are used in the reduced form, which must subsequently be restored because although these analogs are significantly cheaper than their natural counterparts, stoichiometric addition would still not be economically viable. 

As far as chemical regeneration is concerned, the reducing agent sodium dithionite is also widely used to regenerate the artificial cofactors, but the problem remains of the toxicity such chemical has on enzymes. For this reason, alternative ways of regeneration were also sought in this case.

Ishibashi et al. [95], Amao et al. [86], and Ikeyama et al. [94], for example, used photochemical regeneration in the presence of TEOA as an electron donor. Although this type of regeneration performed on the cofactor analog rather than the natural cofactor seems to be easier and more efficient, the need to include several molecules in the system, such as the photosensitizer and an electron donor, makes the final separation difficult [63].

**Figure 7:**
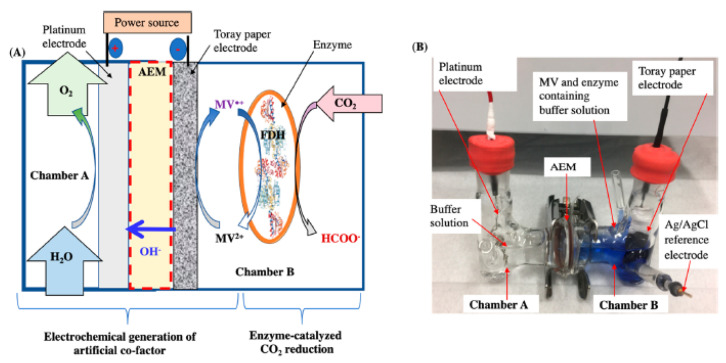
Coupled system for CO_2_ reduction and artificial cofactor electrochemical regeneration. (**A**) Reactor configuration; (**B**) Experimental set-up. Adapted with permission from Ref. [84]. Copyright © 2018 American Chemical Society.

The best regeneration method in terms of both efficiency and ease of industrial application and cost is electrochemical regeneration. Zhang et al. [87] and Jayathilake et al. [84] used electrochemical regeneration: with this approach, only electrons are consumed, and it is possible to eliminate both the photosensitizers and reducing agents. Jayathilake achieves a formic acid yield with MV^+^ regeneration of 97% in 30 h (Figure 7).

Zhang, on the other hand, identified DA^2+^ as the best performing of the various artificial cofactors tested, allowing a formic acid concentration of 3.5 mM to be reached in 1 hour. Moreover, by comparison with the dithionite-reduced and visible-light-reduced artificial cofactors, Zhang showed that electrochemically reduced cofactors have advantages for enzymatic reduction, implying the high potential of electrochemically driven enzymatic reduction of CO_2_ to formic acid.

### 3.4. Cofactor Free Use of the Cascade f Reactions

Considering that the cost of the cofactor and its regeneration is what has the greatest economic impact on the reaction, the possibility of exploring new ways to eliminate the need for it altogether is very attractive. Indeed, using electrochemical direct electron injection would not require the cofactor NADH and minimize the diffusion-induced overpotentials and simplify the product separation [58].

**Figure 8:**
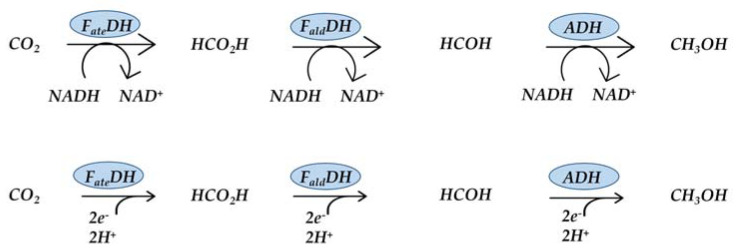
Reaction of CO_2_ reduction to methanol with and without cofactor [51].

Electrochemical techniques make it possible for electrons to pass directly from the electrode to the enzyme, as proposed by Schlager et al. in 2016 (Figure 8) for the reduction of CO_2_ to methanol [51]. The authors used a carbon felt electrode modified with alginate containing the three dehydrogenases in a CO_2_-saturated system without any sacrificial coenzyme. With this system, they managed to obtain about 0.15 ppm methanol and a faradaic efficiency of 10% [51].

**Figure 9:**
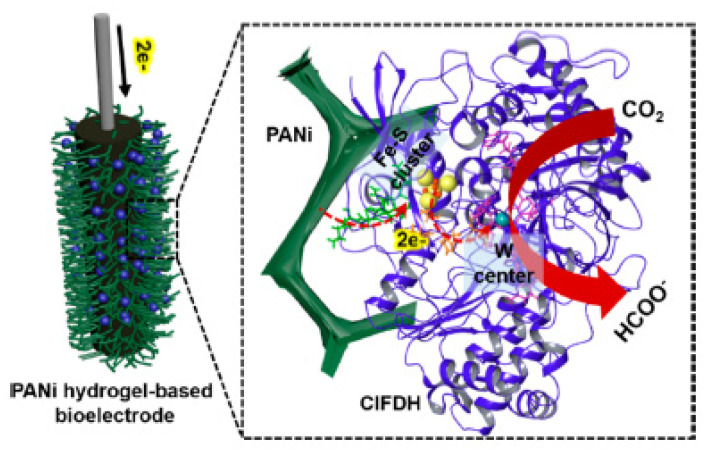
Schematic Illustration of *ClFDH*-PANi Electrode and Direct Electron Transfer from Conductive PANi Hydrogel to *ClFDH* for Electroenzymatic CO2 Conversion to Formate. Reprinted with permission from Ref. [96]. Copyright © 2022 American Chemical Society.

Kuk et al. immobilized the *F_ate_DH* enzyme from *Clostridium ljungdahlii* on a conductive polyaniline (PANi) hydrogel (Figure 9). This enzyme appears to be the one with the highest catalytic efficiency of all known *F_ate_DH* enzymes with a *K_cat_*/*K_m_* = 183 mM^−1^ s^−1^. The hydrogel, being conductive itself, acts as an electrode and, when tested in the reduction of CO_2_ to formic acid, enables a conversion rate of 1.42 μmol h^−1^cm^−2^ and a faradaic efficiency of 92.7% [96].

Another interesting case is the one reported by Seelajaroen et al. [58], in which graphene carboxylic acid is directly joined to the three enzymes, which are subsequently immobilized on carbon felt electrode with an alginate hydrogel matrix. The result of this test yields about 20 ppm of methanol, which corresponds to a 12% faradaic efficiency [58].

Finally, we also reported the case of Alvarez-Malmagro et al., who studied the reduction of CO_2_ to formate by direct electron transfer to *Formate dehydrogenase* from *Desulfovibrio vulgaris* chemically immobilized on modified gold and low-density graphite electrodes, achieving a formate yield of 3.5 µM and a faradaic efficiency of about 100% [61].

However, these systems are quite simple and can improve the stability of enzymes as well as their activity because they couple their immobilization with a direct electron exchange system. There are limitations to the application of such methods due essentially to the small surface area of the electrode and thus the limited availability of the enzyme and low production of methanol. The operational stability times are also quite low; in fact, the reactions in the various cases examined are studied for short times.

### 3.5. Coupling of Immobilization and Regeneration Methods: The Results

The cascade reaction from CO_2_ to methanol is an extensively studied reaction for its potential applicative interest: many steps were taken to overcome the limitations reported above. Various methods of immobilizing enzymes were explored and combined with various methods of regenerating both natural and artificial cofactors. Some of the most recent and interesting cases reported in the literature are listed in Table 4.

In Cazelles’ article [18], it can be seen that the methanol moles by switching from production with free enzymes to that with immobilized enzymes combined with regeneration increases by around 70 times. Zhu et al. [53] found that combining regeneration with co-immobilization of enzymes and cofactor can increase the yield by about three times compared to enzyme immobilization alone. Aresta et al. [67] managed to obtain 100 to 1000 moles of methanol from a single mole of NADH by combining co-immobilization of the three *dehydrogenases* in hybrid alginate beads and photochemical regeneration. Among the most recent is the work of Zhang [59], in which, with respect to a concentration of 0.061 mM of methanol using free enzymes, arrived at 0.742 mM with the combination of regeneration and immobilization; thus, an approximately 12-fold increase in the product was obtained.

The examples above show how the combination of enzyme co-immobilization with cofactor regeneration significantly changes the amount of product that can be obtained, positively and significantly modifying reaction yields, showing the path to potential exploitation.

## 4. Conclusions and Future Perspectives 

The cascade of reactions studied in this review is very interesting as a process for using CO_2_, considered as a resource from which to obtain a product of interesting economic value such as methanol, helping to alleviate the problem of global warming and climate change. This review examined the enzymatic reduction reaction from CO_2_ to CH_3_OH in its various aspects. By starting with the analysis of the properties of the three enzymes involved, the weak points (life of enzymes, amount of cofactor used) of the reactions cascade were discussed, and the existing bottlenecks were highlighted for its up-scale. Much progress has been made so far, especially in finding solutions for the immobilization of enzymes and for cofactor recycling in order to reduce the cost, moving towards cofactor replacement or elimination.

However, despite such efforts, the way to industrial application of this cascade of reactions is still a long one. Nevertheless, the high selectivity (100%) and the fact that water is used instead of H_2_ and the reaction occurs at room temperature make the reaction cascade very attractive.

Enzymes are very expensive and unstable species. Therefore, it will be necessary to work by focusing on immobilization, which, as we have seen, brings positive results in terms of life and productivity. The support must respond to a set of properties in order to avoid enzyme deactivation. The use of MOFs seems to be promising for developing stable and usable heterogenized enzymes. At the same time, genetic engineering could be useful to modify the enzymes in order to improve the catalytic activity, even if MGOs and their products are not accepted in all countries.

If cofactor NADH is used, one must aim for simple systems for regeneration so as to achieve a 100% selectivity directed towards the active form of the cofactor, 1,4-NADH, working with simple systems and avoiding the use of complex, multicomponent reagents that make difficult the post process isolation and increase the production cost. The enzymatic regeneration is the most used but has high costs and increases the complexity of the system. The chemical regeneration is invasive as it may deactivate the enzymes. The electrochemical and the photochemical routes appear to be of interest, supposed that the active isomer, 1,4-NADH, is produced and not the inactive 1,6-NADH or dimers. The alternative is to avoid the use of cofactors, finding new techniques for protons and electrons injection.

## Figures and Tables

**Table 1:** *Km* values for the CO_2_ reduction–formate oxidation reactions.

Enzyme	Reaction	*Km*	Ref.
*F_ate_DH*	CO_2_ → HCO_2_^−^	30–50 mM	[18]
	HCO_2_^−^ → CO_2_	0.5 mM	[18]

**Table 2:** *Km* values for the formate reduction–formaldehyde oxidation reactions.

Enzyme	Reaction	*Km*	Ref.
*F_ald_DH*	HCO_2_^−^ → HCOH	n.d	[18]
	HCOH → HCO_2_^−^	0.09 mM	[18]

**Table 3:** *Km* values for the formaldehyde reduction–methanol oxidation reactions.

Enzyme	Reaction	*Km*	Ref.
*ADH*	HCOH → CH_3_OH	6 mM	[18]
	CH_3_OH → HCOH	100 mM	[18]

## Data Availability

Not applicable.

## Abbreviations

***ADH***, *Alcohol dehydrogenase*; **ALG-SiO_2_**, alginate-silica; **AMV**, 1-aminoethyl-1′-methyl-4,4′-bipyridinium salt; **ATCN-DSCN**, thiophene-modified double shell hollow g-C_3_N_4_ nanospheres; **BPs**, bipyridines; ***CA***, *Carbonic anhydrase*; **CCG-IP**, chemically converted graphene- isatin-porphyrin; **Cc-PAA**, cobaltocene poly(allylamine); **CF**, carbon felt; **CMV**, 1-carboxymethyl-1′-methyl-4,4′-bipyridinium salt; **CV**, 1-carbamoylmethyk-1′-methyl-4,4′bipyridinium iodide; **DA^2+^**, 1,1′-diaminoethyl-4-4′-bipyridinium salt; **DAV**, 1,1′-diaminoethyl-4,4′-bipyridinium salt; **DB**, 1,1′-ethylene-2,2′-bipyridinium bromide; **DC^2+^**, 1,1′-diacarboxymethyl-4,4′-bipyridinium salt; **DCV**, 1,1′-dicarboxymethyl-4,4′- bipyridinium salt; **DEMM**, Disordered Enzymes/ZIF-8 in membrane; ***DH***, *Diaphorase*; **DM**, 1,1′-dimethyl-2,2′bipyridinium chloride; **DV**, 1,1′-dicarbamoylmethyl-4,4′-bipyridinium diiodide; **ECMS**, Enzymes/Coenzyme/ZIF-8 in Solution; **EDTA**, ethylenediaminetetraacetic acid; **EMS**, Enzymes/ZIF-8 in Solution; ***F_ald_DH***, *Formaldehyde dehydrogenase*; ***F_ate_DH***, *Formate dehydrogenase*; ***GCDH***, *Glucose dehydrogenase*; ***GDH***, *Glutamate dehydrogenase*; **GHGs**, Greenhouse Gases; **MA^2+^**, 1-methyl-1′-aminoethyl-4,4′-bipyridinium salt; **MC^2+^**, 1-methyl-1′-carboxymethyl-4,4′-bipyridinium salt; **MCF**, mesostructured cellular foam; **MGOs**, genetically modified organisms; **MOFs**, metal organic frameworks; **MV^2+^**, 1,1′-dimethyl-4,4′-bupyridinium salt or methylviologen; **NAD^+^**, nicotinamide adenine dinucleotide oxidized form; **NADH**, nicotinamide adenine dinucleotide reduced form; **NEMBP**, 1-nicotinamidethyl-1′-methyl-4,4′-bipyridinium salt; **NPS**; nanoparticles; **OECMM**, Ordered Enzymes/Coenzyme/ZIF-8 in membrane; **OEMM**, Ordered Enzymes/ZIF-8 in membrane; **PANi**, polyaniline; **PBS**, Phosphate buffered saline; **PCET**, proton-coupled electron transfer; **PS**, polystyrene; ***PTDH***, *Phosphite dehydrogenase*; **PVDF**, poly(vinylidene fluoride); **QB^2+^**, 1,1′-tetramethykene-2,2′-bipyridinium bromide; **SDT**, sodium dithionite; **TB^2+^**, 1,1′-trimethylene-2,2′bipyridinium bromide; **TEOA**, triethanolamine; **TOF**, turnover frequencies; **Tris-HCl**, tris(hydroxymethyl)aminomethane hydrochloride; **UV**, ultraviolet; ***XDH***, *Xylose dehydrogenase*; ZnTPyPBr, zinc 5,10,15,20-tetra(4-pyridyl)-21H,23H-porphine tetrabromide.
